# Overexpression of the *AtSHI* Gene in Poinsettia, *Euphorbia pulcherrima*, Results in Compact Plants

**DOI:** 10.1371/journal.pone.0053377

**Published:** 2013-01-07

**Authors:** M. Ashraful Islam, Henrik Lütken, Sissel Haugslien, Dag-Ragnar Blystad, Sissel Torre, Jakub Rolcik, Søren K. Rasmussen, Jorunn E. Olsen, Jihong Liu Clarke

**Affiliations:** 1 Bioforsk - Norwegian Institute for Agricultural and Environmental Research, Ås, Norway; 2 Department of Plant and Environmental Sciences, Faculty of Science, University of Copenhagen, Frederiksberg, Denmark; 3 Department of Plant and Environmental Sciences, Norwegian University of Life Sciences, Ås, Norway; 4 Palacky University, Olomouc, Czech Republic; Cankiri Karatekin University, Turkey

## Abstract

*Euphorbia pulcherrima,* poinsettia, is a non-food and non-feed vegetatively propagated ornamental plant. Appropriate plant height is one of the most important traits in poinsettia production and is commonly achieved by application of chemical growth retardants. To produce compact poinsettia plants with desirable height and reduce the utilization of growth retardants, the *Arabidopsis SHORT INTERNODE* (*AtSHI*) gene controlled by the cauliflower mosaic virus *35S* promoter was introduced into poinsettia by *Agrobacterium-*mediated transformation. Three independent transgenic lines were produced and stable integration of transgene was verified by PCR and Southern blot analysis. Reduced plant height (21–52%) and internode lengths (31–49%) were obtained in the transgenic lines compared to control plants. This correlates positively with the *AtSHI* transcript levels, with the highest levels in the most dwarfed transgenic line (TL1). The indole-3-acetic acid (IAA) content appeared lower (11–31% reduction) in the transgenic lines compared to the wild type (WT) controls, with the lowest level (31% reduction) in TL1. Total internode numbers, bract numbers and bract area were significantly reduced in all transgenic lines in comparison with the WT controls. Only TL1 showed significantly lower plant diameter, total leaf area and total dry weight, whereas none of the *AtSHI* expressing lines showed altered timing of flower initiation, cyathia abscission or bract necrosis. This study demonstrated that introduction of the *AtSHI* gene into poinsettia by genetic engineering can be an effective approach in controlling plant height without negatively affecting flowering time. This can help to reduce or avoid the use of toxic growth retardants of environmental and human health concern. This is the first report that *AtSHI* gene was overexpressed in poinsettia and transgenic poinsettia plants with compact growth were produced.

## Introduction

The ornamental industry is one of the fastest growing industries worldwide, especially in Japan and China. Global production of ornamental potted plants and cut flowers comprises about 50 billion €, corresponding to an estimated global consumption between 100 and 150 billion € [1], [2]. The market for cut flowers and potted ornamental plants is not only determined by producers’ choices but also by a continuously growing demand for novelties and high quality [3], [4]. Compaction of plants is one of the most important traits in many ornamental potted plants, e.g. poinsettia [2].

*Euphorbia pulcherrima* Willd. Ex Klotzsch, poinsettia, is a non-food, non-feed and vegetatively propagated ornamental plant, known as a contemporary symbol of Christmas in many parts of the world [5]. It is a short day plant and flowering is initiated when the day length is shorter than a critical length [6]. Generally, poinsettia has an elongated natural growth habit. Dwarf characteristics can be obtained either by directly using dwarf cultivars or by grafting cultivars on dwarf rootstocks [7]. Similarly, spraying with growth retardants such as CCC (chlormequat chloride) or alar (dimethylaminosuccinamic acid), that among others inhibit the biosynthesis of the plant hormone gibberellin (GA), results in compact ornamental potted plants [8]. However, growth retardants are expensive, time consuming to apply and have negative impact on human health as well as the environment. Moreover, the growth regulators will likely be banned in EU countries in the near future [9]–[11].

In the poinsettia industry, alternative strategies like manipulation of temperature, light quality and light duration have previously been tested to control elongation growth of poinsettias [12]–[15]. In northern areas short term diurnal temperature drops, obtained by opening vents in the morning, are commonly used to reduce shoot elongation. However, in warmer periods and warmer areas of the world this is not applicable. Furthermore, in poinsettia phytoplasma is introduced to induce free-branching and this can also result in compact growth [16]. However, the phytoplasma is lost upon exposure of the plant materials to heat treatment as well as meristematic and somatic embryogenesis tissue culture, which is commonly used to obtain disease free plants by removing pathogens such as the poinsettia mosaic virus (PnMV) [16].

Genetic engineering is increasingly adopted as an important alternative to conventional breeding [1], [17]. Recently, it was shown that introduction of the *Arabidopsis thaliana SHORT INTERNODES (SHI*) gene into *Kalanchoë* and *Populus* resulted in dwarfed growth without any morphological abnormalities [18], [19]. However, in *Populus* the dwarfing effect on the stem was only very weak, although the internode and petiole lengths were significantly reduced. On the other hand, overexpression of *GA2-oxidase* genes, which control GA inactivation, resulted in dwarfed plants with delayed flowering time in *Solanum* and *Arabidopsis* [20], [21]. Also, antisense silencing of the GA biosynthesis gene *GA20-oxidase* resulted in smaller leaves, delayed flowering time and reduced fertility in *Arabidopsis* [8]. Overexpression of the *Arabidopsis* GA signalling gene *GIBBERELLIN INSENSITIVE* (*GAI*) in apple and *Chrysanthemum* reduced plant height, but was correlated with reduced rooting ability and delayed flowering in the respective species [7], [22]. In light of these observations, introduction of the *AtSHI* gene to poinsettia might be highly interesting as a means to control elongation growth without introducing undesired morphological or developmental changes. *SHI* gene is a plant specific transcription factor belonging to the *SHI* gene family, and it has been identified in different plants species like tomato, rice, soybean and *Medicago truncatula* [23]. The *Arabidopsis SHI* gene family consists of 10 members; *SHI, STYLISH 1 (STY1)* and *STY2, LATERAL ROOT PRIMORDIUM 1 (LRP1)* and *SHI-RELATED SEQUENCE 3* to *8 (SRS3* to *SRS8)* [18], [23]–[25]. These corresponding proteins have two highly conserved regions, a RING-like zinc finger motif positioned in the N-terminal end and an IGGH domain of unknown function in the C-terminal part of the protein [23], [25], [26].

All genes could be amplified from all tissues of *Arabidopsis*, except *SRS8*, indicating it might be a pseudogene [25]. STY1 is the closest paralog of SHI having two identical domains in the N and C terminal. The *SHI* family genes have both diverse and redundant functions in plant growth and are involved in shoot apical region development as well as flower and leaf development [23], [25], [26]. In these respects *SHI/STY*-related genes appear important in gynoecium development, vascular formation and organ identity in floral whorls two and three [25]. It has been documented that *SHI* and *STY* are expressed in the apical region of the developing gynoecium [25], [27]–[29]. Overexpression of *SRS7* conferred dwarfed growth in *Arabidopsis* with small and curled leaves, anther dehiscence and abnormal floral development [30].

SHI family members act as DNA binding transcription activators and may act on plant growth and development by affecting phytohormones like auxin and GA, which among other things control shoot elongation in response to different stimuli [31]–[35]. Plants overexpressing *SHI* are dwarfed, but a normal, more elongated phenotype can be restored by application of auxin [27], [28]. Also, the *YUCCA4* (*YUC4*) auxin biosynthesis gene is induced by the SHI/STY family proteins. However, SHI and STY appear to differ slightly in function as shown by lower affinity of SHI than STY1 to the *YUC4* promoter in the yeast two-hybrid system [36]. Application of indole-3-acetic acid (IAA) to the apical meristem has been shown to increase biosynthesis of bioactive GA [37]–[39]. *SHI* overexpressing *Arabidopsis* plants show increased levels of the inactive GA_34_ compared to wild type plants [24]. Furthermore, in *Brassica SHI-*related genes have been identified as negative regulators of GA-induced cell division [40].

In this study, we report for the first time successful use of genetic engineering as a tool to control elongation growth in poinsettia, which is among the economically most important potted ornamental plants worldwide. Compact growth was obtained by overexpressing the *AtSHI* gene by using a recently developed *Agrobacterium-*mediated transformation method for poinsettia [41]. Apart from the desired dwarfed growth habit, no developmental abnormalities were scored and flowering time was unaffected.

## Materials and Methods

### Plant Materials

Poinsettia (*Euphorbia pulcherimma* Willd. ex Klotzsch) cv. Millenium cuttings were grown in the greenhouse under 16 h photoperiod provided by high pressure sodium (HPS) lamps (400W, GAN 4-550, Gavita, Superagro, Andebu, Norway) at 21±2°C with an average relative air humidity (RH) 70±5%. For *Agrobacterium tumefaciens*-mediated transformation 5–15 mm long internode stem explants were excised from 8–10 weeks old poinsettia plants. The explants were surface sterilized with 70% ethanol (1 min), 1% NaOCl (10 min) and then rinsed thoroughly three times with sterile deionized and autoclaved water for 3, 10 and 20 min. After sterilization, stem segments (1–1.5 mm thickness) were excised and utilized for *Agrobacterium* transformation.

### Transformation, Selection and Plant Regeneration

**Figure 1:**
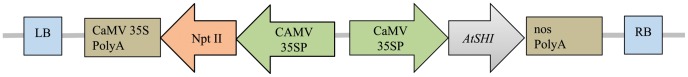
Gene construct: pKanIntron-35S-*SHI* used for *Agrobacterium*-mediated transformation of poinsettia.

Plasmid vector pAt35S:SHI was constructed and introduced into *Agrobacterium tumefaciens* strain GV3850 as previously described in details by Lütken et al. [19]. A brief schematic drawing of the *SHI* gene expression cassette is shown in Figure 1. The *Agrobacterium* culture and subsequent transformation were carried out basically as described by Clarke et al. [41]. The stem segments were inoculated in the *Agrobacterium* suspension for 10 min with gentle shaking. The stem segments were then blotted on sterile filter paper and transferred to callus induction medium (CIM) (MS medium supplemented with 0.2 mg l^−1^ BAP, 0.2 mg l^−1^ CPA and 30 g l^−1^ sucrose) for co-cultivation up to 72 h in the dark at 24°C. After co-cultivation, the explants were transferred to the CIM medium with antibiotic selection containing 10 mg l^−1^ kanamycin and 400 mg l^−1^ claforan (Aventis Pharma Ltd, Norway) for about 10 days. The embryogenic calli were subsequently transferred after every three weeks to somatic embryo induction medium (SEIM) (MS medium contains 0.3 mg l^−1^ NAA, 0.15 mg l^−1^ 2ip and 30 g l^−1^ sucrose) supplemented with antibiotics 25 mg l^−1^ kanamycin and 400 mg l^−1^ claforan. Shoots and plantlets which derived from the somatic embryos were transferred to root induction (RI) medium (1/2 strength MS with 20 g l^−1^ sucrose) with or without hormones (1 mg l^−1^ IAA or IBA). The pH was 5.7–5.8 in all MS media. Regenerated plants were transferred to soil and grown in a greenhouse once the root system was developed.

Light conditions provided by fluorescent tubes (Philips Master TL-D Super 58W/840, Eindhoven, The Netherlands) for *in vitro* cultures were 23 µmol m^−2^ s^−1^ for callus induction and 30 µmol m^−2^ s^−1^ for the SEIM and RI media, respectively, under a 16 h photoperiod at 24°C.

### PCR Analysis

Genomic DNA was isolated from the putative transgenic poinsettia plants using the DNeasy Plant Mini Kit (Qiagen GmbH, Hilden, Germany) according to the manufacturer’s instructions. PCR reactions (20 µl) were performed using 100 ng DNA, 0.2 µM of each primer, and 2xHotStarTaq Mastermix and supplied water from HotStarTaq® Plus Master Mix Kit (Qiagen GmbH, Hilden, Germany). Primer sequences used to amplify a fragment of approximately 500 bp for the *AtSHI* gene (At5g66350) were 5′-ACTCTAACGCTGACGGTGGA-3′ (forward) and 5′-TGCTGACCGGTAGAAAGCTG-3′ (reverse). PCR amplification was performed in a C1000™ thermal cycler (BIO-RAD, Singapore) using the following conditions: 15 min at 95°C (1 cycle) followed by 30 s at 95°C, 45 s at 56°C, and 1 min at 72°C (35 cycles) with a final extension of 7 min at 72°C (1 cycle). PCR products were analysed by ultraviolet light after electrophoresis on 0.8% (W/V) agarose gels.

### Southern Blot Analysis

Total genomic DNA was isolated from the leaves of the WT control plants and the PCR positive transgenic poinsettia lines using the DNeasy Plant Maxi Kit (Qiagen GmbH, Hilden, Germany). Southern blot analysis was performed according to Sambrook et al. [42]. Ten micrograms of genomic DNA were digested with *Hind*III for 5 h and separated on 1% (W/V) TBE agarose gel overnight at 37 V, and subsequently transferred onto Genescreen Plus(™) Hybridization Transfer membrane (NEF 988001 PK, Boston, MA, USA). The 500 bp PCR product representing the coding region of the *AtSHI* gene as described above was used as a probe for hybridization. Membranes were hybridized with the ^32^P-labelled probe overnight at 65°C. After hybridization the membranes were washed and then exposed to film as described by Clarke et al. [41].

### RNA Isolation and cDNA Synthesis

Young leaves of transgenic poinsettia and control plants were harvested for RNA extraction with E.Z.N.A Plant RNA Mini Kit (Omega bio-tek, Norcross, GA, USA) according to the manufacturer’s instructions. RNAs were subsequently treated with Turbo DNA-*free*™ kit (Ambion Inc., Austin, TX, USA) to eliminate DNA contamination. RNA quality and quantity were evaluated using agarose gel electrophoresis and Nanodrop 2000 Spectrophotometer (Wilmington, Delaware, USA), respectively. Two micrograms total RNA from each sample was used to synthesize cDNA in a 20 µl reaction using the cDNA SuperScript® VILO™ synthesis kit from Invitrogen (Carlsbad, CA, USA) according to the manufacturer’s instructions.

### Real-Time Quantitative PCR

Real- time quantitative PCR analysis was performed in a 25 µl reaction volume, using 2.5 µl twentyfold diluted cDNA as a template with 12.5 µl of 1x Power SYBR® green PCR master mix (Applied Biosystems, Warrington, UK) and 0.4 µM of each primer. Primers were designed using Primer3 online software and sequences are listed in Table 1.

**Table 1:** Primers for real-time PCR expression analysis of *AtSHI* in poinsettia.

Target gene	Forward primer (5′-3′)	Reverse primer (5′-3′)	Product size
*AtSHI*	AGCTATGGCAACACCCAAAC	ATCCAGCCTTTGTTGCTGTT	71
α-tubulin	TGGAGCTCTCTTTGCTTCAA	CCAACAAAGCTGCATAGCAA	81

Reactions were conducted in an Applied Biosystems 7900HT Real-time PCR system combined with SDS 2.3 version software (Applied Biosystems, Singapore). The PCR conditions were as follows: 50°C for 2 min and 95°C for 10 min, followed by 40 cycles of 95°C for 15 s, 60°C for 1 min. Dissociation curve analysis was carried out to verify the specificity of the PCR amplification. PCR efficiencies were calculated from a dilution series of genomic DNA of transgenic plants for each primer pair of target gene and endogenous gene by following the equation E = 10^−1/slope^. There were three replications for all samples including the control samples except the TL2, and a no template control was included for each primer pair. The transcript level was estimated by threshold cycle (C_t_) values of each sample. The differences of C_t_ values between the endogenous *α*–tubulin and the target gene were normalized using the formula 2^−ΔΔCt^ [43]. The relative transcript levels (fold-differences) of the genes were converted to scale log_10_ values.

### Phenotypical Analysis of Transgenic Lines (*AtSHI*) Compared to Control Plants

The independent transgenic lines, TL1–6 from TL1, TL2, TL3 and control plants were vegetatively propagated. After root formation, the plants were potted in 13 cm plastic pot filled with *Sphagnum* peat (Veksttorv, Ullensaker Almenning, Nordkisa, Norway). The plants were kept at 21±2°C, 70±5% RH in the greenhouse under a 16 h photoperiod (8 h darkness) at an irradiance of 150±25 µmol m^−2 ^s^−1^ provided by HPS lamps (400W, GAN 4–550). The plants were pinched over 3–4 leaves allowing three shoots to grow per plant. Four plants from each transgenic line including WT control plants were transferred to growth chambers (controlled environment) without any natural light. Light was provided by General Electric company, Fairfield, CT, USA at an irradiance of 100±20 µmol m^−2 ^s^−1^ (measured by LI-COR Quantum/Radiometer/Photometer, Model LI-250, Lincoln, Nebraska, USA) under a 10 h photoperiod for flowering. The temperature was 21°C and the RH was 70±5%.

The plants were watered daily during the growth period with commercial nutrient solutions. The side shoots developed during the growth period were removed and counted and only three shoots were allowed to grow per plant. The length of these three shoots was measured from the base of the stem to the shoot apical meristem every fourth day until flowering. At the end of the experimental period (after flowering) petiole length of four mature leaves was measured. The number of leaves and bracts (namely the transition leaves which formed red color more than 40%) were counted and the average internode lengths were calculated by dividing the shoot height by the number of leaves and bracts. Relative chlorophyll content was measured from the middle leaf of the three side shoots on each plant by a chlorophyll content meter (Model CL-01, Hansatech instruments, Norfolk, England). Leaf and bract areas were measured by an area meter (Model 3100 area meter, LI-COR, Lincoln, Nebraska, USA). Specific leaf and bract area (SLA and SBA, respectively) were each determined from area/dry weight. After recording the fresh weight, the dry weight was recorded after drying at 65°C until a constant mass was reached. The number of days until visible bracts and cyathia was counted. At the selling stage plants were moved to a postharvest test room to compare differences in cyathia abscission and bract necrosis. The climate in the test room was 21°C, 30–40% RH and an irradiance of 10 µmol m^−2 ^s^−1^ was provided 12 h daily by fluorescent tubes (Philips Master TL-D 58W/830).

Three replicate experiments in growth chambers were carried out in which phenotypic observations were performed, samples for auxin analysis collected and postharvest quality tested. In addition to the growth chamber experiments, two to four plants from each line were grown in the greenhouse with conditions as described above for evaluation of the morphological performance of transgenic plants under long day conditions (16 h). Both short and long day treatment experiments were conducted during November 2011 through January 2012.

### Auxin Measurements

In the growth chamber experiment, elongating shoot tips were harvested after three weeks of starting short day conditions and immediately placed in liquid N_2_. The samples were freeze-dried using a freeze dryer machine (Heto Holten A/S, Allerød, Denmark). For each genotype, 3 replicate samples, each containing 3 shoot tips (elongating parts of the stem) were used for auxin analysis. The frozen plant materials were ground in a mortar and extracted for 5 min with 1 ml cold phosphate buffer (50 mM, pH 7.0) containing 0.2% sodium diethyldithiocarbomate. ^15^N and ^2^H_5_-labeled internal standards of indole -3-acetic acid (IAA) and IAA metabolites were obtained from OlChemlm (Olomouc, Czech Republic). The measurements were performed in duplicate. The samples were processed according to Pěnčík et al. [44] using high performance liquid chromatography (HPLC) coupled to a tandem triple-quadrupole mass spectrometer (MS/MS).

### Statistical Analysis

Different growth parameters of transgenic and WT control plants were subjected to analysis of variance (General Linear Model procedure) and Tukey’s pair wise comparison test (p≤0.05) using Minitab version 16 (Minitab Inc., State College, PA, USA).

## Results

### PCR Screening and Southern Blot Analysis of Transgenic Poinsettia Plants

**Figure 2:**
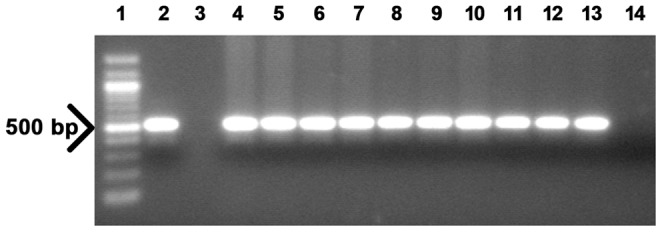
PCR analysis of poinsettia transformed with the *AtSHI* gene (genomic DNA was extracted from leaves). Lane 1∶100 bp marker, lane 2: plasmid control, lane 3: blank, lanes 4–11: eight plants from independent transgenic lines 1 (TL1) (individuals 1–8), lane 12: TL2, lane 13: TL3 and lane 14: WT control line. The arrow indicates the 500 bp band.

**Figure 3:**
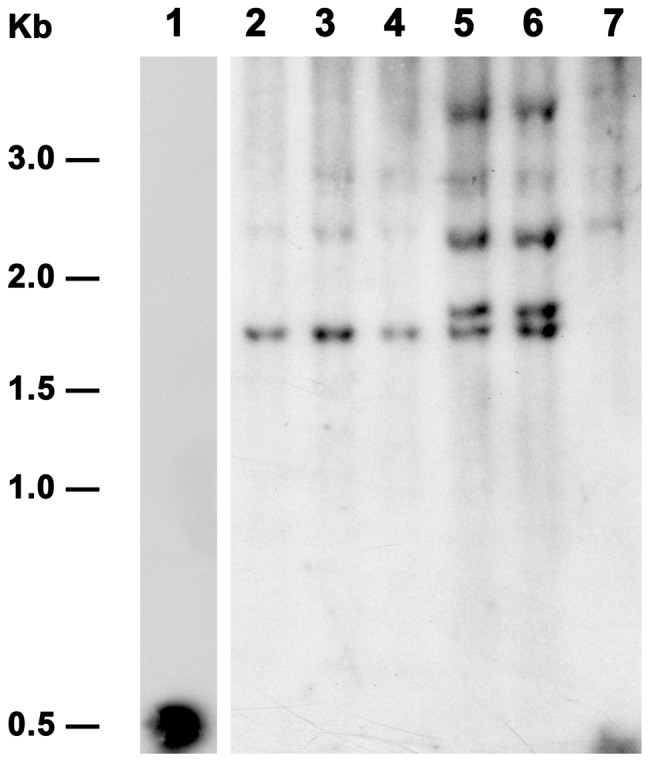
Southern blot analysis of PCR positive transgenic poinsettia lines overexpressing the *AtSHI* gene. *Hind*III-digested total genomic DNAs were hybridized with a *AtSHI* probe (the 500 bp PCR product). Lane 1: positive control, the 500 bp PCR product; lane 2–4: TL1–3, TL1–4, TL1–6 of the transgenic line one (TL1); lane 5: TL2; lane 6: TL3, lane 7: WT control.

To generate stable compact growth transgenic poinsettia plants by overexpressing the *AtSHI* gene, *Agrobacterium*-mediated transformation experiments were carried out using stem segment explants. After selection and regeneration through somatic embryogenesis, regenerated poinsettia plants were obtained and established in the greenhouse. Using *AtSHI* specific primers, genomic DNA of putative transgenic poinsettia plants was screened by PCR analyses for presence of *AtSHI*. Three independent transgenic lines were confirmed, one plant was selected for each line except the transgenic line one (TL1) of which eight plants from the same clone were used (Figure 2). Transgenic lines were further analyzed by Southern blot hybridization. Results of Southern blot analysis confirmed the stable integration of transgene into the poinsettia genome (Figure 3). Of the three transgenic lines analyzed, line TL1 (with three individual transgenic plants) showed single copy of the transgene whereas TL2 and TL3 with one transgenic plant each showed two copies of the transgene integration (lanes 2–4, 5 and 6 in Figure 3). Lane 7 is the WT negative control, whereas lane 1 is the positive control.

### *AtSHI* Expression in the Transgenic Lines

**Figure 4:**
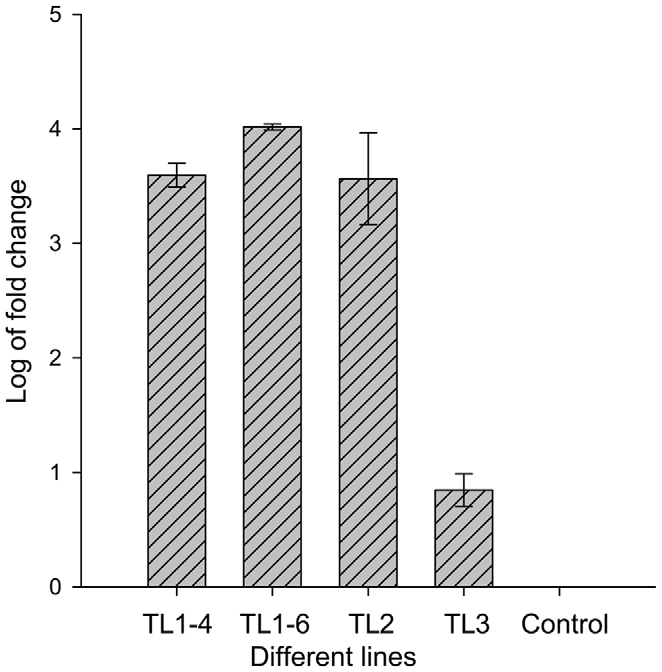
Quantitative real-time PCR analysis of *AtSHI* transgene in different transgenic lines of poinsettia. Two microgram total RNAs from transgenic poinsettia lines and the endogenous control, α-tubulin gene were used for synthesize cDNAs prior the real-time qPCR analysis. Values are means of three technical replications except TL2 (n = 2). Data were analyzed using the 2^−△△C^_T_ method and represented as log_10_ values. Vertical bars represent the ± SE (standard error).

*AtSHI* transcript levels were analysed by real-time quantitative PCR in *AtSHI*-expressing transgenic poinsettia lines and the WT controls. Three transgenic lines (TL) were analyzed. Of these, two plants (TL1–4 and TL1–6) were from TL1, while TL2 and TL3 with one plant each were included for the quantitative real time PCR. *AtSHI* transcript levels varied among the transgenic lines as shown in Figure 4. The highest relative levels of transcript were found in TL1–6, whereas the lowest expression was found in TL3.

### Reduced Growth Elongation without Change in Flowering Time and Keeping Quality in *AtSHI* Expressing Transgenic Lines

**Figure 5:**
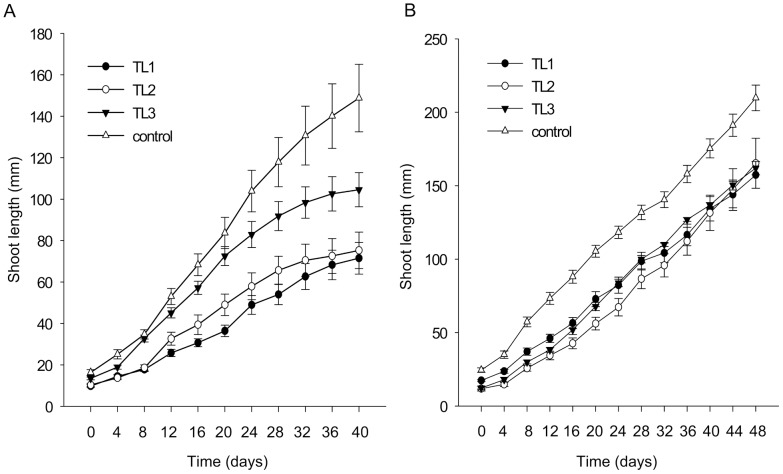
Height comparison among the different transgenic lines (TL) of *AtSHI* overexpressing poinsettia and untransformed control plants grown under short day (10 h) (A) and long day conditions (16 h) (B). Vertical bars represent the ± SE (standard error), n = 11–12 and 6–12 in A and B, respectively.

**Figure 6:**
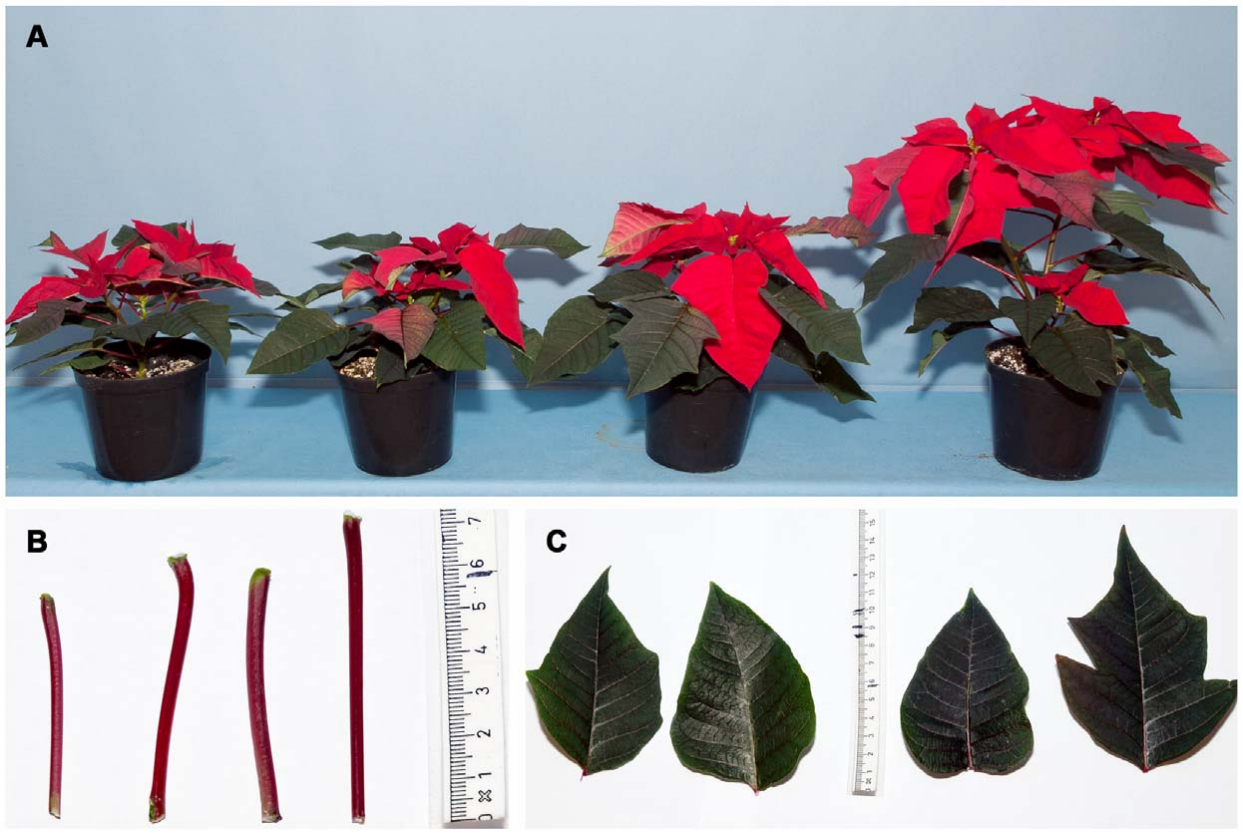
Transgenic *AtSHI* overexpressing poinsettia plants (A), petioles (B) and leaves (C). In each figure: from the left different transgenic lines; TL1, TL2, TL3 and non-transformed control plants are shown. The plants were grown at 21±2°C, a 10 h photoperiod at an irradiance of 100±20 µmol m^−2 ^s^−1^.

Over-expression of *AtSHI* in poinsettia significantly reduced plant height compared to WT control plants. When grown under short day (SD) conditions, the TL1 transgenic line showed the strongest height reduction response (52%) whereas the TL2 and TL3 plants were reduced in height by 49% and 30%, respectively, compared to WT control plants (Figures 5A, 6A). Under long day (LD) conditions, the height reduction was 25%, 21% and 23% in TL1, TL2 and TL3, respectively, compared to control plants (Figure 5B). No differences were observed in leaf color or leaf shape, and there was no significant difference in petiole length (Figure 6 B, C).

**Figure 7:**
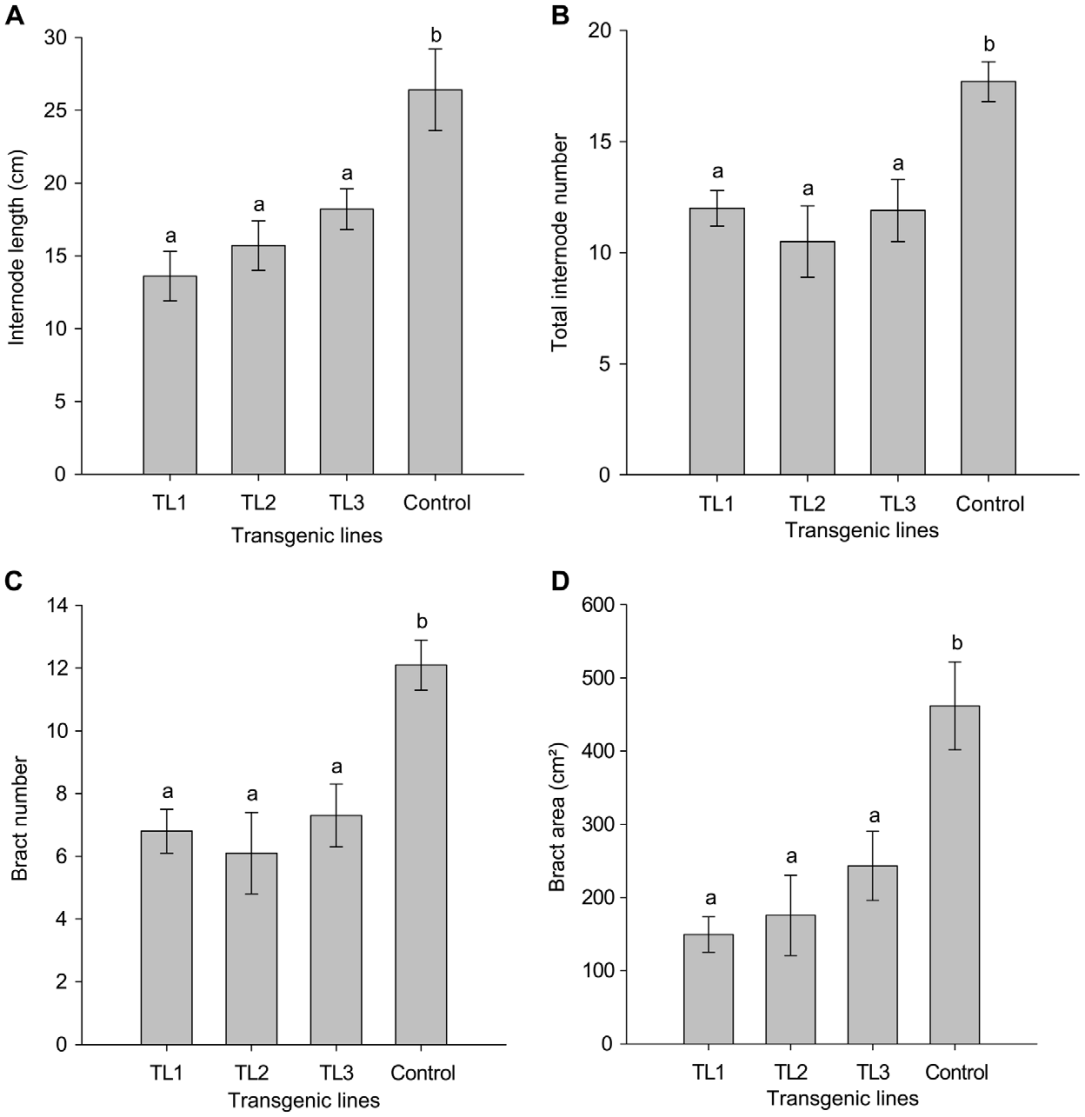
Effects of *AtSHI* overexpression on internode length (A), total internode number (sum of bracts and leaves) (B), bract number (C) and bract area (D) of different transgenic lines and control plants of poinsettia. Plants were grown under short day conditions of a 10 h photoperiod, n = 11–12. Mean values with different letters are significantly different based on ANOVA followed by a Tukey’s test at p≤0.05. Vertical bars represent the ± SE (standard error).

As investigated under SD conditions, overexpression of the *AtSHI* gene significantly reduced the internode lengths and the internode number compared to WT control plants (Figure 7A, B). On average, internode lengths were significantly reduced by 49%, 41% and 31% and the internode number by 32%, 41% and 33% for TL1, TL2 and TL3, respectively. The transgenic plants had significantly lower bract number and reduced bract area as shown in Figure 7C and D. The average bract number was reduced by 44%, 50% and 40% and the bract area by 68%, 62% and 47% for TL1, TL2 and TL3, respectively. In TL1 the total dry weight of stems, leaves and bracts, specific leaf area, total leaf area and shoot diameter differed significantly from the WT control plants (Table 2). No significant difference in relative chlorophyll content was found between the transgenic and control plants (Table 2).

**Table 2:** Comparison of growth parameters among transgenic (T) lines of poinsettia overexpressing the *AtSHI* gene, TL1, TL2, TL3 and control plants under short day conditions of a 10 h photoperiod in a controlled environment.

	Line TL1	Line TL2	Line TL3	Control
Height (mm)	71.5±7.7 b	75.3±8.8 b	104.5±8.2 b	148.8±16.3 a
Relative chlorophyll content	27.7±1.0 a	28.0±2.1 a	25.5±2.1 a	24.6±2.2 a
Shoot diameter (mm)	10.2±0.6 b	12.0±1.1 a	13.9±0.3 a	13.2±0.3 a
Total leaf area (cm^2^)	199.8±17.3 c	247.6±32.3 bc	385.1±29.0 a	341.0±27.0 ab
Specific leaf area (cm^2^ g^−1^)	283.3±3.7 a	271.9±6.6 ab	265.1±5.8 b	262.7±5.0 b
Total dry weight (g)	1.2±0.1b	1.8±0.3 ab	2.7±0.3 a	2.7±0.3 a

Different letters within a parameter indicate significant differences (Tukey’s test at p<0.05). n = 11–12. Mean value ± SE (standard error) are given.

Bract formation started after four weeks when plants were kept under SD conditions and visible cyathia were observed after 5 weeks. No significant difference in time to initiation of flowering was observed between the transgenic and control plants. The development of bract color formation appeared a bit faster in control plants compared to transgenic plants. Bract necrosis was not observed and no significant differences in cyathia abscission or keeping quality between the transgenic plants and control plants were observed (data not shown). In the postharvest room, the cyathia abscission started after three weeks and one week later about 90% of the bracts were abscised from all plants (data not shown).

### Reduced Height and Internode Lengths Correlate with Reduced IAA Levels

To investigate the mechanism of the reduced plant height and internode lengths in *AtSHI* overexpressed plants, the levels of IAA were investigated in shoot tips of transgenic poinsettia lines as well as the WT control plants. In the transgenic plants the IAA levels showed a reduction of 11–31% compared to the WT control plants as shown in Table 3. The lowest IAA levels (31% less) were recorded in the transgenic line TL1.

**Table 3:** Endogenous levels of auxin and their metabolites (pmol g^−1^ DW) in transgenic lines and control plants of poinsettia grown under a short photoperiod of 10 h.

Line	IAA	IAA-Asp	IAA-Glu	Total auxin
Transgenic lineTL1	2004.8±14.2 a	169.9±23 a	99.6±7.5 a	2274.3±35.3 a
Transgenic lineTL2	2575±104 a	240.3±29.7 a	89.4±4.0 ab	2905±127 a
Transgenic lineTL3	2290±371 a	171.8±6.3 a	62.63±4.6 b	2524±375 a
WT control	2892±328 a	204.3±10 a	98.19±9.59 a	3194±340 a

Different letters within a parameter indicate significant differences (Tukey’s test at p≤0.05). Mean value ± SE (standard error) are given; Three shoots of one plant were treated as one replicate and three separate replicates were analyzed.

## Discussion

In this study we have demonstrated that the over-expression of the *AtSHI* gene is an efficient tool to reduce plant height in the economically highly important ornamental plant poinsettia. PCR analysis verified the *AtSHI-*expressing transgenic poinsettia lines, while Southern blot analysis further confirmed the transgene integration into the poinsettia genome. With the vegetatively propagated nature of poinsettia, the desired compact growth characteristic in the transgenic poinsettia lines over-expression of the *AtSHI* gene will be maintained and propagated by cuttings, a clear advantage over sexually propagated plants. Recently, a dwarfing effect of this gene was shown also in *Kalanchoë* and *Populus* [18], [19]. *SHI* gene has among others been suggested to act to control contents of plant hormones involved in control of shoot elongation [27], [31], [36], [45].

### *AtSHI* Expression in Poinsettia Results in Reduced Internode Elongation

*AtSHI* gene expression resulted in a significant reduction of shoot height compared with the control plants under SD as well as LD conditions (Figures 5, 6A). The strongest shoot length reductions were observed in TL1, which compared to control plants showed 52% and 25% reduction under SD and LD, respectively. In all three transgenic lines, the reduction in shoot lengths was more pronounced under SD compared to LD. This could be ascribed to a generally more vigorous height growth under the higher light sum of the LDs of 16 h. Under LD plants were vegetatively growing whereas under SD conditions shoot elongation ceased due to floral induction. The overexpression of *AtSHI* in poinsettia is comparable with observations made in *Populus, Kalanchoë* and *Arabidopsis* [18], [19] where it also significantly reduced the height. In this study, the largest reduction in internode length in transgenic poinsettia was 49% compared to WT control plants.

In our current study, the number of internodes was reduced in the *AtSHI* overexpressed transgenic poinsettia plants. Such information is available from neither *Kalanchoë* nor *Arabidopsis*, which is a rosette plant and does not have elongated internodes, but the result is in contrast to the results from *Populus* [18], [19]. On the other hand, the petiole length was reduced in the *Populus* [18], [19]. In poinsettia, bract number and bract area were reduced significantly in the transgenic plants compared to the control plants. A reduction of 47 to 67% in bract area was observed in the different transgenic lines (Figure 7D). The reduced bract size may have impact on the ornamental value of poinsettia. About 41% higher total leaf area and 8% lower specific leaf area were observed in the transgenic line TL1 compared to control plants (Table 2). Leaf colour and alterations were visually observed where the transgenic lines did not show any differences from the control plants. In Figure 6C, both serrate lobed and non-serrate lobed leaves are present. We have observed both types of leaves in the same plants of three transgenic lines and the control plants. Thus, there was no difference in leaf shape of transgenic and control plants. Fridborg et al. [24] observed darker green leaves in *Arabidopsis*, whereas Lütken et al. [19] did not observe any differences in leaf colour in *Kalanchoë* but *AtSHI* was over-expressed in both plants. The overexpression of *AtSHI* caused pleiotropic changes during the developmental stages of *Arabidopsis*. The reason for differences of phenotypic characteristics might be due to the different habits of growth and flowering stage of *Arabidopsis* and *Kalanchoë* as well as poinsettia. Life span and vegetative stage are very short in *Arabidopsis* compared to *Kalanchoë* or poinsettia. No significant difference in relative chlorophyll content was observed among the transgenic lines and control plants. This might suggest that the *AtSHI* overexpression does not reduce photosynthesis in plants. Rather, the relative chlorophyll mean value was higher in the transgenic lines compared to the WT control plants. However, 56% total dry weight in TL1 was significantly lower compared to the WT controls. About 23% reduction in shoot diameter in TL1 was observed compared to the control plants. This result is similar to *SHI* overexpressing *Kalanchoë*, but in contrast to *Arabidopsis* [19], [24]. There was no significant difference of branching among the transgenic *AtSHI* poinsettias and control plants (data not shown). This is in contrast to the *shi* mutant in *Arabidopsis,* where more branches were observed in *SHI* overexpressing plants [24].

### Reduced Elongation Growth Correlates with Reduced IAA and Higher Expression Levels of *AtSHI*

The reduced stem extension of the plants expressing the *AtSHI* gene was correlated with reduced endogenous levels of IAA levels (Tables 2, 3), with the lowest levels in the shortest lines (TL1). SHI/STY family members regulate plant development. In *Arabidopsis* the *STY1* interacts with the promoter of the auxin biosynthesis gene *YUC4* and induces its transcription [36], [46]. YUC family proteins act as rate limiting enzymes of the tryptophan-dependent auxin biosynthesis pathways [47], [48]. In *Arabidopsis* it was shown that the SHI/STY family controls the developmental process through regulation of auxin biosynthesis [27]. Our results with reduced height and reduced IAA levels in poinsettia overexpressing *AtSHI* are similar to those obtained in a previous study of the moss *Physcomitrella patens* [27]. Two genes of *PpSHI* reduced elongation and reduced endogenous auxin levels in this moss [45]. The *Arabidopsis* auxin mutant (*ettin-1*) is affected by *SHI/STY* family mutants [27], [49]. It is also reported that elongation involves cell division and cell elongation due to cell wall modification activated by auxin among others [50]. Elongation is also well known to be related to GA levels or sensitivity to GA [20], [51], [52]. However, it has been reported that IAA promotes the biosynthesis of active gibberellin (GA) and that auxin transport inhibitors reduce the active GA content in pea and *Arabidopsis* [37], [38], [53], [54]. This reduction of shoot length and internode length is also consistent with results obtained in the moss *P. patens* [45]. The transgenic moss lines showed reduced levels of auxin presumably related to the overexpression of *PpSHI*.

In *AtSHI* overexpressing lines of *Populus*, the internode number and the concentration of cytokinin were increased. The height and number of shoot apical meristems (SAM) was reduced due to reduced content of cytokinins [55]. The auxin level was also lower in transgenic plant tissue. This supports the *Arabidopsis* data where auxin levels and biosynthesis were reduced by the introduction of the *SHI* family member gene *sty1* [27], [36]. In contrast, *AtSHI* expressing *Populus* did not show any change in auxin concentration, but the cytokinin levels were decreased [18]. No significant difference was observed in the petiole length in poinsettia and a similar result was obtained in *Arabidopsis.*

The *AtSHI* transcript levels correlated well with the observed phenotype. Plants that contained relatively high levels of the *AtSHI* transcript were severely dwarfed, whereas less dwarfed plants contained lower transcript levels (Figures. 4, 5A).

### Flowering Time and Keeping Quality are Not Affected in the *AtSHI* Overexpressing Transgenic Poinsettia Plants

In the ornamental industry, the most important feature is flowering. The attractive part of poinsettia is the bract colour (formation of red anthocyanin pigment in the transition leaves) formation, which was observed at nearly the same time in both control and transgenic plants. The completion of bract colour formation was a little bit faster in control plants compared to transgenic plants. This is quite similar to previous studies, where no difference in flowering time was found in *sty1–2* and wild type *Arabidopsis* plants [46]. Multiple inputs like photoperiod, light quality and GA converge to regulate flowering [56], [57]. GA promotes flowering in some LD plants like *Arabidopsis* and *Lolium* and inhibits flowering in SD plants such as rice [58], [59]. However, GA’s involvement in floral initiation is complex and varies from species to species [60], [61]. The bract formation was visible after four weeks when plants were kept under SD conditions. Cyathia abscission was observed in the postharvest room under standard conditions. The cyathia abscission started after three weeks and at the end of 4 weeks about 90% bracts were abscised in both transgenic and WT control plants (data not shown). Furthermore, no negative effects were seen on cyathia abscission or bract necrosis or the postharvest quality due to *AtSHI* overexpression in poinsettia. In *Arabidopsis* a negative effect of *SHI* overexpression is late flowering, but this was not observed in either *Kalanchoë* or in our study of poinsettia [19].

### Conclusions

The economic importance of poinsettia provides a driving force to improve this important ornamental plant by using genetic engineering. With respect to control of plant morphology, this method is time saving, convenient and environmentally friendly compared to conventional breeding and application of hazardous chemical growth retardants. We have here demonstrated that compact poinsettia plants without delay in flowering or change in keeping quality can be obtained by using ectopic *AtSHI* expression as a tool. For poinsettia, dwarf characteristics with good keeping quality are required for high ornamental and market value. Dwarf plants are also convenient for handling and transportation compared to more elongated plants, and need less space in expensive production facilities. Although there is generally, especially in Europe, a negative attitude towards genetic engineering of food crops which are consumed by humans, the attitude might be less negative for plants grown for other purposes such as non-food, non-feed ornamental plants. Hence, the use of *Agrobacterium*-mediated transformation to introduce *AtSHI* into commercially grown poinsettia cultivars can be very promising in the poinsettia industry, being environmentally friendly, beneficial to the economy and to human health by avoiding hazardous effects of plant growth retardant application. Due to the vegetative propagation nature of poinsettia, the acquired dwarfing effect and other desirable characteristics will be stably inherited in vegetative cuttings used in propagation.
