# Correction: Overexpression of the *AtSHI* Gene in Poinsettia, *Euphorbia pulcherrima*, Results in Compact Plants

**DOI:** 10.1371/annotation/010f6c7f-9745-4810-a370-c96fb1f583e9

**Published:** 2013-07-16

**Authors:** M. Ashraful Islam, Henrik Lütken, Sissel Haugslien, Dag-Ragnar Blystad, Sissel Torre, Jakub Rolcik, Søren K. Rasmussen, Jorunn E. Olsen, Jihong Liu Clarke

An affiliation for the first author was not indicated. M. Ashraful Islam is affiliated with institution 1, Bioforsk - Norwegian Institute for Agricultural and Environmental Research, Ås, Norway, as well as institution 3, Department of Plant and Environmental Sciences, Norwegian University of Life Sciences, Ås, Norway. 

